# Clarity in Reporting Multiple Long‐Term Conditions in Research: The Need for Consistency and Consensus

**DOI:** 10.1111/dom.70804

**Published:** 2026-04-20

**Authors:** Mark P. Funnell, Ash Routen, Lauren L. O'Mahoney, Deborah Ikhile, Kamlesh Khunti, Patrick J. Highton

**Affiliations:** ^1^ Diabetes Research Centre University of Leicester, Leicester General Hospital Leicester UK; ^2^ NIHR Applied Research Collaboration East Midlands, Diabetes Research Centre University of Leicester Leicester UK

**Keywords:** condition clusters, multimorbidity, multiple long‐term conditions, outcomes, reporting

## Abstract

Multiple long‐term conditions (MLTC), also known as multimorbidity, affects a substantial proportion of the global population and represents an increasing challenge to healthcare systems, individuals and society. Despite increasing attention and investment, progress in MLTC research is hindered by conceptual inconsistencies and a lack of standardised reporting. Challenges include varied terminology, differing definitions of chronicity and substantial variation in the number and type of conditions included across studies. These inconsistencies contribute to differences in prevalence estimates and reduced comparability across the literature. Moreover, many clinical trials involve participants living with MLTC but do not collect or report co‐existing conditions, leaving their experiences largely invisible within research and limiting applicability of findings for this population. Heterogeneity in disease clusters and outcome selection further complicates evidence synthesis. In this commentary, we highlight that improving the clarity and impact of MLTC research requires transparent reporting of MLTC status, the use of consensus‐based condition lists, identification of clinically and socially meaningful clusters and prioritisation of outcomes aligned with what matters most to people living with MLTC (e.g., independence, treatment burden and quality of life). Improving reporting practices is needed to generate evidence that improves care, informs policy and enhances the wellbeing of people living with MLTC.

## 1 Background

Multiple long‐term conditions (MLTC), also referred to as multimorbidity, is defined as the co‐existence of two or more long‐term conditions. In England, approximately 8.8 million people (14.8% of the population) live with MLTC [1]. A similar burden of MLTC has been observed in low‐ and middle‐income countries (LMICs) [2, 3], and global prevalence is expected to increase markedly as populations age and socioeconomic inequalities persist. The National Institute for Health and Care Research (NIHR) has identified MLTC as a strategic research priority [4], while both Lord Darzi's report on the state of the National Health Service (NHS) in England [5] and the NHS 10‐Year Plan [6] highlight the growing burden of MLTC on patients and services.

To address this growing challenge, a major initiative on MLTC was initially started by the Academy of Medical Sciences to summarise existing evidence and identify research gaps [7]. In continuation of this initiative, UK research funders, including the NIHR, Medical Research Council (MRC) and UK Research and Innovation (UKRI), have invested substantially, coordinating efforts through a cross‐funder framework on MLTC launched in 2020 [8]. Moreover, the NIHR established the MLTC Cross‐NIHR Collaboration to unite infrastructure and accelerate research efforts in MLTC [9]. Despite such initiatives, research in this area continues to face difficulties, partly due to differing definitions, heterogeneity of included conditions, outcome selection and a lack of clear reporting. This commentary highlights conceptual and reporting issues in MLTC research and offers guidance for future research to improve reporting clarity and consistency.

## 2 Definitional and Conceptual Uncertainty

One of the first challenges in MLTC research is definitional. The Academy of Medical Sciences and World Health Organization (WHO) define MLTC as the co‐existence of two or more long‐term conditions, which may be physical, mental, or infectious [7, 10]. This definition is widely used and recognised, although alternatives exist. For instance, The European General Practice Research Network (EGPRN) offers a broader conceptualisation, defining MLTC as any combination of chronic disease with at least one other disease (acute or chronic) or a biopsychosocial or somatic risk factor [11].

A proliferation of terms, including MLTC, multimorbidity, co‐morbidity, multicondition, polymorbidity, polypathology and multipathology, complicates the field further. MLTC is a newer term, increasingly preferred by people living with MLTC, reflecting their desire for language that is less medicalised and more person‐centred [12]. This shift in terminology highlights the importance of aligning research with patient perspectives, ensuring that the field develops in a way that resonates with those affected.

Inconsistency also arises from the conditions included in MLTC research. A study of ~1.75 million people in Scotland, which included 40 conditions, reported a prevalence of 23.3% [13]. A more recent Delphi study recommended that 24 conditions should always be included in MLTC research, and 35 should usually be included unless strong justification exists otherwise [14]. Applying this consensus framework, the prevalence of MLTC across England's ~60 million population was estimated at 14.8% [1]. Notably, only 24 conditions overlapped between the Scottish and English studies, highlighting the impact of differing inclusion criteria on prevalence estimates.

While consensus frameworks represent an important step towards standardisation of condition lists, it is important to recognise that the aforementioned Delphi study [14] was based on a selective expert panel, with limited representation of experts and public contributors from LMICs. Therefore, the generalisability of the proposed condition lists across diverse settings and populations may be limited. Consensus‐based condition lists should be considered a starting point for research rather than a definitive framework, with flexibility to allow for context‐specific conditions relevant to specific populations or research questions. Furthermore, MLTC extends beyond the presence of conditions, with social and psychological factors having an important role in the development, progression and lived experiences of people with MLTC [1].

In LMICs, MLTC research is further complicated by the need to consider both communicable and non‐communicable diseases [15]. Communicable diseases, such as human immunodeficiency virus (HIV) and tuberculosis, remain prevalent in some LMIC settings and often co‐exist with rising non‐communicable disease burdens, producing distinctive MLTC clusters such as HIV, hypertension and diabetes [15]. These clusters differ from those observed in high‐income countries (HICs), where the burden of non‐communicable diseases dominates and many communicable diseases have been successfully controlled.

Further complexity arises from how chronicity itself is defined. MLTC prevalence in England varied widely, from 41% to 74%, depending on whether one or multiple diagnostic codes over time were used to define a long‐term condition [16]. Together, competing definitions, inconsistent terminology and differing condition inclusion and timeframes have fragmented the evidence base, complicated cross‐study comparisons and have made it difficult to synthesise data and assess the true scale and impact of MLTC.

## 3 Research Invisibility

Many studies that focus on a single index condition often include participants with MLTC, typically without collecting data on co‐existing conditions or describing the study population as living with MLTC. For example, large cardiovascular or diabetes trials often report that a high percentage (e.g., > 70%) of participants have at least one additional long‐term condition, yet trial design, outcomes, reporting and interpretation remain focused on the index condition. This creates an invisibility of MLTC within research, limiting understanding of interventions in real‐world populations living with MLTC and complicating evidence synthesis. Recent evidence synthesis work has highlighted this issue, showing that it is often unclear whether trial populations include people living with MLTC and, if so, which conditions are represented [17]. Such uncertainty makes it difficult to pool results and draw definitive conclusions, ultimately having knock‐on effects for future MLTC research.

## 4 Cluster Heterogeneity

Individuals living with MLTC present with highly variable combinations of conditions. In England, more than 205 000 unique combinations have been reported among those aged over 80, with the likelihood of having four or more conditions increasing drastically with age [1]. Such heterogeneity complicates research synthesis, intervention design and outcome selection.

To identify dominant disease clusters, researchers have used a range of statistical approaches (e.g., factor analysis, hierarchical clustering, unified clustering algorithms, multiple correspondence analysis and network‐based methods) [1, 18, 19, 20], however, findings differ depending on the method used and the extent to which chance associations are accounted for [21]. Nevertheless, certain MLTC clusters emerge consistently. For instance, cardiometabolic conditions such as diabetes, hypertension and cardiovascular disease often co‐exist due to shared metabolic, vascular and behavioural risk factors. MLTC clusters change across the life course; younger adults are more likely to present with combinations involving asthma and mental health (e.g., depression and anxiety), whereas in older adults cardiometabolic and musculoskeletal clusters [1] are dominant. Moreover, MLTC clusters may differ by ethnicity [22]. Ethnic minority groups experience a disproportionate burden of MLTC but remain underrepresented in research [22, 23], limiting our understanding of this intersection.

## 5 Measuring What Matters

The diversity of MLTC clusters makes outcome selection difficult. Research has historically focused on single diseases, often excluding people with MLTC from trials to protect internal validity, resulting in findings with low external validity that poorly reflect real‐world populations. Moreover, trials typically measure disease‐specific intermediate endpoints (e.g., HbA1c in diabetes, blood pressure in hypertension), but these outcomes often overlook what matters most to people living with MLTC (e.g., maintaining independence, reducing treatment burden, satisfaction with care and accumulating additional conditions). For example, in an individual living with cardiometabolic MLTC (e.g., cardiovascular disease, type 2 diabetes and hypertension), clinically meaningful outcomes may include functional status and independence, treatment burden, chronic kidney disease progression and major adverse cardiovascular events. These outcomes may better reflect the complexity of managing multiple conditions and the priorities of people living with MLTC.

Quality of life is often chosen as a primary outcome because of its holistic relevance, but trials often rely on generic patient‐reported outcome measures (PROMs), such as EQ‐5D‐5L or SF‐36. These PROMs, while widely validated, lack sensitivity and validity in heterogeneous MLTC populations [24]. PROMs tailored to MLTC populations, such as the Multimorbidity Questionnaire (MMQ1), which has recently been translated into English language and validated for broader use, offer a way to capture these lived experiences but remain underused [25]. Nevertheless, both generic and MLTC‐specific PROMs have limitations, including ceiling effects and limited sensitivity in heterogeneous MLTC populations. Without consensus on core outcomes, evidence synthesis remains incoherent, limiting impact. Recent work has begun to address this issue; for instance, the COSMOS study developed core outcome sets for MLTC prevention and treatment in LMICs, highlighting outcomes such as quality of life, adverse events, adherence to treatment and out‐of‐pocket costs as essential for consistent reporting [26]. Greater alignment between outcome selection and patient priorities, alongside the continued development of MLTC‐specific outcome sets, will be essential to improve the relevance and impact of future research.

## 6 Recommendations for Future Research

Advancing research on MLTC requires consistent definitions, transparent reporting and prioritisation of outcomes that reflect the needs of people living with MLTC. To achieve this, where possible, research should report the following checklist (Box 1) as a minimum reporting standard. Researchers should use the checklist and report according to study design; for example, for randomised controlled trials, this includes MLTC definition, distribution of MLTC burden and MLTC status using condition lists and, where possible, stratification of outcomes by MLTC burden; whereas for observational research, studies should describe how conditions were identified (e.g., code lists or phenotypes), data sources and the ascertainment periods and clustering methods used.

BOX 1 **Minimum reporting checklist for MLTC research.**

1. *MLTC Definition*: Provide the definition of MLTC used, specify the criteria for MLTC (e.g., ≥ 2 or ≥ 3 conditions) and cite the relevant reference(s).
2. *Condition Lists*: Use consensus‐based condition lists as a default starting point, while allowing flexibility for context‐specific conditions relevant to specific populations or research questions (e.g., including chronic infectious diseases for research in LMICs [15]). Report and justify any deviations from consensus lists (e.g., study setting or disease burden) and consider sensitivity analyses comparing consensus‐based and study‐specific lists where feasible.
3. *Distribution of MLTC Burden*: Systematically describe patients with MLTC, including the proportion of patients affected and by which conditions.
4. *Data Source and Identification Method*: Describe data sources used (e.g., electronic health records, surveys) and explain how conditions were ascertained, such as through diagnostic codes, clinical assessments, or self‐report.
5. *Chronicity Definition*: Specify the time frame used to identify conditions and how chronicity was defined (e.g., two disease codes within a 6‐month time period). Consider how different time windows may affect MLTC prevalence and reclassification across age, ethnic and socioeconomic groups. Additionally, consider whether the included conditions are acute or chronic and ensure this aligns with the definition of MLTC applied in the study.
6. *Outcomes*: Clearly define primary and secondary outcomes, indicate whether patient‐reported, clinical, or administrative and specify alignment with established core outcome sets where possible.
7. *Cluster Methods*: Studies using clustering approaches should clearly describe which conditions were included and how they were encoded, the clustering method and key parameters used, how the number of clusters was determined and whether findings were robust and reproducible.

## 7 Summary

Addressing MLTC is an urgent healthcare challenge, but current research is hindered by conceptual inconsistencies, underreporting and fragmented outcome measures. By systematically capturing MLTC status, including consensus‐based conditions, identifying meaningful clusters and prioritising patient‐relevant outcomes, research will become more coherent and efficient, generating evidence that better guides policy, improves care and enhances the quality of life for people living with MLTC.

## Author Contributions

M.P.F.: design, writing manuscript – original draft, project administration. A.R., L.L.O. and D.I.: writing manuscript – review and editing. K.K. and P.J.H.: design, writing manuscript – review and editing.

## Funding

The authors have nothing to report.

## Conflicts of Interest

The authors declare no conflicts of interest.

## Data Availability

Data sharing not applicable to this article as no datasets were generated or analysed.
